# Platelet bioenergetics correlate with skeletal muscle respiration in a murine model of type II diabetes

**DOI:** 10.3389/fmolb.2025.1639882

**Published:** 2025-11-14

**Authors:** Mia S. Wilkinson, Emily J. Ferguson, Justin Bureau, Roan A. L. Haggerty-Goede, Dalia M. Miller, Michelle Kuriakose, Jennifer L. M. H. Veeneman, Patricia D. A. Lima, Chris McGlory, Kimberly J. Dunham-Snary

**Affiliations:** 1 Department of Medicine, Queen’s University, Kingston, ON, Canada; 2 School of Kinesiology and Health Studies, Queen’s University, Kingston, ON, Canada; 3 Department of Biomedical and Molecular Sciences, Queen’s University, Kingston, ON, Canada; 4 Queen’s CardioPulmonary Unit, Queen’s University, Kingston, ON, Canada

**Keywords:** platelets, skeletal muscle, bioenergetics, mitochondria, metabolism

## Abstract

Mitochondrial bioenergetic research in skeletal muscle is limited by the need for biopsies. We executed a proof-of-concept study to evaluate whether blood platelets could serve as a minimally invasive surrogate for skeletal muscle mitochondrial respiration in mice. Using Seahorse extracellular flux analysis, platelet respiration was measured in healthy C57BL/6J and leptin receptor-null *db/db* mice, while high-resolution respirometry (Oroboros O2k) assessed mitochondrial function in white gastrocnemius muscle of the same animals. A critical component of this study was extensive methodological optimization for platelet bioenergetics analysis in mice. We provide comprehensive methodological details and guiding principles for performing Seahorse bioenergetic assays on mouse platelets. Our foundational findings also suggest platelet mitochondria can reflect tissue-level mitochondrial health, pointing to a potential “liquid biopsy” approach for assessing metabolic status. Multiple key metrics of respiration showed significant correlations between platelets and muscle in the same animals, indicating that platelet bioenergetic profiles mirror the metabolic status of skeletal muscle in healthy and genetically diabetic mice. This work lays the conceptual and methodological foundation for future studies in human metabolic diseases where muscle bioenergetic dysfunction is implicated but current methods are not implementable for clinical surveillance. This study provides foundational proof-of-concept in healthy and diabetic mice, motivating validation in human studies as the next step toward biomarker development and precision medicine strategies.

## 1 Introduction

Cardiometabolic disease (CMD) is highly prevalent worldwide, with obesity alone responsible for *annual* healthcare costs of $173 billion in the United States (Noubiap et al., 2022; Smolderen et al., 2010; Ward et al., 2021). CMD, or metabolic syndrome, is diagnosed by the ≥3 of the following factors: abdominal obesity (population-/country-specific definitions); elevated triglycerides (≥150 mg/dL); reduced HDL-c (<40 mg/dL, males; <50 mg/dL, females); hypertension (systolic ≥130 and/or diastolic ≥85 mmHg); elevated fasting glucose (≥100 mg/dL) (Alberti et al., 2009). This high-risk metabolic state increases cardiovascular disease (CVD) and Type II diabetes (T2DM) risk, leading causes of morbidity and mortality worldwide, with heart diseases accounting for one-third of all deaths, globally (IHME, 2025). Rising CMD incidence highlights the insufficiency of diet, exercise, and current treatments to address underlying genetic and biomolecular factors impacting disease etiology (Sakakibara et al., 2019). A critical step in the progression from CMD to CVD and T2DM is the emergence of insulin resistance, resulting in metabolic disturbances and compromised tissue function (Petersen et al., 2004; Stump et al., 2006). Skeletal muscle is the largest insulin-sensitive tissue in the body and exhibits functional decline during progression of CMD, CVD, and T2DM (Petersen et al., 2004; Stump et al., 2006; Kennel et al., 2015; Tallis et al., 2018). Mitochondria, a hub of cellular metabolism, play a fundamental role in CMD-related skeletal muscle insulin resistance and metabolic inflexibility through reduced oxidative capacity, increased reactive oxygen species (ROS), and impaired coupling (Stump et al., 2006; Asmann et al., 2006; Lowell and Shulman, 2005; Romanello and Sandri, 2015).

The study of skeletal muscle mitochondrial respiration has emerged as a translational tool for characterizing mitochondrial function in human pathology (Chacko et al., 2014; Dranka et al., 2011; Hill et al., 2012). Skeletal muscle mitochondria can serve as the ‘canary in a coal mine’ to provide early warning of bioenergetic crisis for determining the severity and progression of complex and multifactorial diseases (Chacko et al., 2014). While skeletal muscle mitochondrial bioenergetic dysfunction is recognized as a major player in age- and disease-associated decline in skeletal muscle health (Romanello and Sandri, 2015; Kelley et al., 2002; Petersen et al., 2003), both a unifying mechanism and targeted treatments are lacking; this is, in part, due to the requirement of a skeletal muscle biopsy for bioenergetic assessment. Muscle biopsies are invasive (Dengler et al., 2014; Sutu et al., 2024; Bonafiglia et al., 2020; Scal et al., 2006), require local anesthetic (which can introduce complications including allergic reaction, etc. (Schatz, 2025; Jenerowic et al., 2014)), can sometimes be complicated in diabetic patients (Quinlan et al., 2021), and are difficult to scale clinically for large populations in the context of routine clinical monitoring (Dengler et al., 2014; Bonafiglia et al., 2020; Scal et al., 2006; Quinlan et al., 2021; Wilkinson and Dunham-Snary, 2023; Wilson et al., 2018; Nishikawa et al., 2021). These factors, combined with the patient perception that muscle biopsy may be painful (Dengler et al., 2014; Sutu et al., 2024; Bonafiglia et al., 2020; Scal et al., 2006), create a bottleneck for research, hindering characterization of pathology-associated mitochondrial dysfunction and limiting the clinical utility of tissue bioenergetics as a biomarker. The emergence of platelet bioenergetics as a surrogate for skeletal muscle mitochondrial function removes these barriers (Braganza et al., 2020; Chacko et al., 2013; Petrus et al., 2019). Indeed, peripheral blood platelets, the cell fragments of megakaryocytes, have been shown to reflect skeletal muscle mitochondrial metabolism in animal models and humans (Braganza et al., 2019; Rose et al., 2019; Tyrrell et al., 2016). The comparative ease of obtaining platelets, their abundance, highly metabolic phenotype, and high responsiveness to environmental changes all support their candidacy as a biomarker of mitochondrial function (Wilkinson and Dunham-Snary, 2023). Moreover, the lack of a nucleus makes platelets an ideal tool for investigating the contribution of mitochondrial DNA to mitochondrial function (Melchinger et al., 2019).

Compared to platelet mitochondria, the relative inaccessibility of skeletal muscle mitochondria necessitates an accessible and reliable method of assessment (Wilkinson and Dunham-Snary, 2023). The translational potential of platelet bioenergetics is being explored across disease states and its suitability as a biomarker is implicated in many disease states with inaccessible primary tissue as the site of pathology. Due to limited control in human studies (i.e., high variability in lifestyle factors), murine models are commonly used to study complex diseases and define mechanisms driving correlations such as that between platelets and skeletal muscle bioenergetics. In this proof-of-concept study, we used genetically diabetic (*db/db*) and healthy C57BL/6J controls, a strain commonly employed for preclinical metabolic research, and optimized a reliable, reproducible mitochondrial bioenergetics assay for platelets. We subsequently examined whether the correlation between platelet and skeletal muscle bioenergetics, previously demonstrated in humans and non-human primates (Braganza et al., 2019; Rose et al., 2019; Tyrrell et al., 2016; Westerlund et al., 2024), extends to mice. Establishing correlation in healthy mice allows for future studies investigating platelet bioenergetics in preclinical models of disease; this method represents a promising ‘liquid biopsy’ that may offer early insights into muscle metabolic health in complex human diseases and improve prognostic and diagnostic biomarker implementation in clinical practice.

## 2 STAR methods

### 2.1 Key resources table

**Table:** 

Reagent or Resource	Source	Identifier
Antibodies		
CD41-APC	Invitrogen	17-0411-82
CD45-AlexaFluor700	Invitrogen	56-0451-82
P-Selectin-PE	Invitrogen	12-0626-80
Chemicals, peptides, and recombinant proteins		
Sodium citrate	BioShop	CIT001.500
Erythrocyte Lysis Buffer	Invitrogen	00-4,300-54
Prostaglandin I_2_	Cayman Chemical	18,220
Oligomycin A	Millipore Sigma	O4876-5 MG
FCCP	Millipore Sigma	C2920-10 MG
Antimycin A	Millipore Sigma	A8674-50 MG
Rotenone	Millipore Sigma	R8875-1G
Rodent Complex IV Enzyme Activity Microplate Assay	Abcam	ab109911
Critical commercial assays		
Mitochondrial Stress Test Kit	Agilent	103015-100
XF DMEM Medium	Agilent	103575-100
Experimental models: Organisms/strains		
C57BL/6J *Mus Musculus* – 6 weeks of age	The Jackson Laboratory	RRID: IMSR_JAX:000664
B6.BKS(D)-*Lepr* ^*db*^/J *Mus Musculus* – 14 weeks of age	The Jackson Laboratory	RRID: IMSR_JAX:00069

### 2.2 Resource availability

#### 2.2.1 Lead contact

Further information and requests for resources and reagents should be directed to and will be fulfilled by the lead contact, Kimberly Dunham-Snary (Kimberly.DunhamSnary@queensu.ca).

#### 2.2.2 Data and code availability

- Data reported in this article are available upon request from the lead contact.
- This paper does not report original code.
- Any additional information required to reanalyze the data reported in this work paper is available from the lead contact upon request.

### 2.3 Experimental model and study participant details

#### 2.3.1 Animals

All animal studies carried out were approved by the University Animal Care Committee at Queen’s University (Protocols 2023-2390 and 2024–2520). Briefly, 4-5-week-old male C57BL/6J mice (n = 30) were purchased from the Jackson Laboratory (Jax) and fed standard rodent diet (5020 – Mouse Diet 9F; LabDiet, St. Louis, MO, United States) and water *ad libitum* for 1–2 weeks until 6 weeks of age; 6 weeks was chosen to ensure minimal age-associated changes in metabolism, redox balance, and inflammation (Dunham-Snary et al., 2018). 10-week-old male B6.BKS(D)-*Lepr* ^*db*^/J (*db/db*; n = 12) mice were purchased from Jax and fed standard rodent diet (5020 – Mouse Diet 9F; LabDiet, St. Louis, MO, United States) and water *ad libitum* for 4 weeks until 14 weeks of age. All animals were anesthetized with 3%–5% isoflurane, subsequently euthanized via cardiac puncture, and cervical dislocation as a secondary means of euthanasia.

### 2.4 Method details

#### 2.4.1 Tissue collection

Animals were anesthetized via induction with isoflurane and euthanized via cardiac puncture. Whole blood was collected into a pre-filled 1 mL syringe containing 3.2% sodium citrate (0.1 mL) for platelet isolation. Gastrocnemius muscle was excised and immediately placed in ice-cold BIOPS buffer (2.77 mM CaK_2_EGTA, 0.5 mM dithiothreitol, 20 mM imidazole, 7.23 mM K_2_EGTA, 50 mM MES hydrate, 6.56 mM MgCl_2_ × 6H_2_O, 5.77 mM Na_2_ATP, 15 mM Na_2_Phosphocreatine, 20 mM taurine; pH 7.2) for skeletal muscle respirometry. Experimental workflow is displayed in Supplementary Figure S1.

#### 2.4.2 Platelet isolation

Whole blood was centrifuged at 250 x *g* for 2 min at room temperature (RT). Platelet-rich plasma (PRP) was collected. Erythrocyte lysis buffer supplemented with PGI_2_ (0.02 mg/mL; “platelet buffer”) was added to whole blood, which was then centrifuged again (250 x *g*; 2 min; RT). The second plasma fraction was collected and combined with the first PRP fraction to maximize platelet yield. PRP was centrifuged (400 x *g*; 5 min; RT) and the supernatant (platelet-poor plasma) was discarded. Pelleted platelets were resuspended in platelet buffer and incubated (as a wash step; 6 min; RT) to facilitate erythrocyte lysis before centrifugation (400 x *g*; 5 min; RT). The supernatant was discarded, and the platelet pellet was resuspended in 50 µL platelet buffer. Platelet count was obtained via Beckman Coulter Counter Z Series, using a 50 μm aperture with the upper and lower limits set to 5.1 fL and 4.3 fL, respectively, to account for the size difference between mouse and human platelets (Corash, 1989). Each sample was measured in triplicate and averaged to obtain a final cell concentration.

#### 2.4.3 Flow cytometry

Platelet samples were isolated from n = 3 mice as described, and isolated cell fractions were divided evenly, with one aliquot of each sample incubated with 1 U/mL thrombin at 37 °C for 15 min. Platelets were washed with fluorescence-activated cell sorting (FACS) buffer (800 x *g*; 5 min; RT), and subsequently incubated with CD41-APC (platelet marker), CD45-AlexaFluor700 (total leukocyte marker), and P-Selectin-PE (platelet activation marker) antibodies in FACS buffer for 30 min at 4 °C. Samples were washed twice with FACS buffer, resuspended in FACS buffer, and analyzed using a SH800 Cell Sorter (Sony Biotechnology). Fluorescence minus one (FMO) controls were used to determine gating and staining quality. Analysis was performed using FlowJo to confirm purity (Supplementary Figure S2) and quiescence (Supplementary Figure S3) of platelets used for respirometry studies.

#### 2.4.4 Murine platelet mitochondrial stress test (XFe24)

Platelet mitochondrial oxygen consumption rate (OCR) was measured via high-resolution respirometry (HRR) using a Seahorse Extracellular Flux Analyzer (XFe24; Agilent Technologies, Santa Clara, California, United States) (Divakaruni et al., 2014). Cell seeding density, oligomycin titration, and FCCP titration were performed to optimize XFe24 assay conditions for mouse tissue. Platelets were seeded at a density of 20 × 10^6^; samples were plated in duplicate when sufficient tissue was available. The plate was centrifuged (250 x *g*; 1 min) in a swing bucket centrifuge (acceleration = 1, zero brake for deceleration), rotated and centrifuged again under the same conditions to ensure even coating of platelets in each well. Seahorse XF DMEM Medium (5 mM HEPES, 15 mM glucose, 2 mM glutamine, 1 mM pyruvate; pH 7.45) was added to each well.

Platelet OCR measurements were first normalized to pre-assay cell seeding density. Basal respiration (OCR_basal_) was recorded (Divakaruni et al., 2014), then measurements were taken following sequential injections of:

1.Oligomycin (2.5 µM) – inhibits ATP synthase, revealing proton leak respiration (OCR_leak_) (Divakaruni et al., 2014).
2. FCCP (0.4 µM) – uncouples the electron transport chain (ETC) producing maximal uncoupled respiration (OCR_max_) (Divakaruni et al., 2014).
3. Antimycin A + Rotenone (5 µM each) – inhibit complexes III and I, respectively, to define non-mitochondrial respiration (OCR_non-mito_), which is then subtracted from all other parameters (Divakaruni et al., 2014).

Raw bioenergetic data are subsequently processed using Equations 1–5 to derive the parameters previously defined:

$${{\text{OCR}}_{\text{non}-\text{mito}}=\text{OCR}}_{\left(\text{post}-\text{Antimycin }A/\text{Rotenone}\right)}$$ (1)

$${{\text{OCR}}_{\text{ATP}}=\text{OCR}}_{\text{basal}}-{\text{OCR}}_{\text{leak}}$$ (2)

$${\text{Reserve}\text{Capacity}\;\left(\text{ResCap}\right)=\text{OCR}}_{\max}-{\text{OCR}}_{\text{basal}}$$ (3)

$$\text{Reserve Capacity }\left(ResCap\%\right)=\frac{{\text{OCR}}_{\max}}{\;{\text{OCR}}_{\text{basal}}}\times 100$$ (4)

$$\text{Coupling Efficiency }\left(CE\%\right)=\frac{{\text{OCR}}_{\text{ATP}}}{{\text{OCR}}_{\text{basal}}}\times 100$$ (5)

One replicate sample had OCR_ATP_, OCR_leak_, and CE (%) data excluded due to technical artifact upon oligomycin injection (i.e., injection of an air bubble).

#### 2.4.5 Permeabilized muscle fiber bundle preparation

White gastrocnemius muscle tissue was placed in ice-cold BIOPS buffer (2.77 mM CaK_2_EGTA, 0.5 mM Dithiothreitol, 20 mM imidazole, 7.23 mM K_2_EGTA, 50 mM MES Hydrate, 6.56 mM MgCl_2_ × 6H_2_O, 5.77 mM Na_2_ATP, 15 mM Na_2_Phosphocreatine, 20 mM Taurine; pH 7.2) (Veksler et al., 1987; Letellier et al., 1992). All visible connective tissue and fat was removed from the muscle sample prior to, and during, mechanical separation. Muscle bundles were formed by gently separating the fibers along the longitudinal axis of the muscle using fine-tipped forceps under a microscope (AmScope, United States). Fiber bundles were weighed in ∼1.5 mL of tared ice-cold BIOPS. Weighing bundles in this manner allowed fiber bundles to remain relaxed and hydrated. Bundle wet weights were obtained in duplicate, and the average of the two values was used to normalize rates of mitochondrial respiration to fiber bundle mass. As such, the rates of respiration reported herein are mass-specific mitochondrial respiration rates. Fiber bundles remained in ice cold BIOPS until chemical permeabilization with the cholesterol-specific detergent saponin (Saks et al., 1998). Muscle bundles were then chemically permeabilized for 30 min in BIOPS with 40 μg/mL saponin at 4 °C. Following permeabilization, bundles were washed in Buffer Z (5 mg/mL fatty acid-free BSA, 1 mM EGTA, 30 mM KCl, 10 mM KH_2_PO_4_, 105 mM K-MES, 5 mM MgCl_2_ × 6H_2_O; pH 7.2) for 15 min at 4 °C (Perry et al., 2011).

#### 2.4.6 Murine skeletal muscle respirometry (O2k)

Mitochondrial OCR was measured via HRR using the Oxygraph-2 k (O2k; Oroboros Instruments, Innsbruck, Austria). All mitochondrial respiration measurements were obtained while the chamber oxygen concentration was between 400-180 μM. All respiration measurements were performed in 2 mL of Buffer Z, at 37 °C with stirring at 750 rpm, in duplicate, and in the presence of the myosin ATPase inhibitor blebbistatin (5 μM), which was added to each chamber prior to bundle insertion and chamber oxygenation (Perry et al., 2011). Data were acquired every 2 s with the uncorrected rate of mitochondrial oxygen consumption (pmol/s/mL) calculated from 40 data points. Raw oxygen consumption values were reported in pmol/s/mL on DatLab and subsequently corrected for bundle wet weight (wt, expressed as pmol/s/mg wet wt). Substrate addition occurred as follows: pyruvate (5 mM), malate (2 mM), glutamate (10 mM), ADP titrations (500 μM, 5 mM, 15 mM), cytochrome c (10 µM), succinate (20 mM), oligomycin (2.5 µM), antimycin A (2.5 µM)/rotenone (0.5 µM).

All respiration rates were first corrected for background fiber bundle oxygen consumption (in the absence of exogenous substrates) and for non-mitochondrial respiration (Rox, measured after antimycin A + rotenone), yielding true mitochondrial flux (Equation 6). Complex I–driven OXPHOS capacity (CI, Equation 7) was measured after addition of complex I substrates + ADP; and combined complex I + II OXPHOS capacity (P, Equation 8) after succinate addition. LEAK respiration (L, Equation 9) was defined as the rate after oligomycin addition. From these, we calculated ATP-linked (Equation 10), CE(%, Equation 11) and RCR (Equation 12) (Chance and Williams, 1955; Brand and Nicholls, 2011):

$${{\text{OCR}}_{\text{corrected}}=\text{OCR}}_{\text{measured}}-{\text{OCR}}_{\text{background}}-\text{Rox}$$ (6)

$${\text{CI}\;\text{OXPHOS}=\text{OCR}}_{\left(\text{CI}\;\text{substrates}+\text{ADP}\right)}$$ (7)

$${P=\text{OCR}}_{\left(\text{CI}+\text{CII}\;\text{substrates}+\text{ADP}\right)}$$ (8)

$${L=\text{OCR}}_{\left(\text{post}-\text{oligomycin}\right)}$$ (9)

ATP−linked=P−L (10)

$$\text{CE}\left(\%\right)=\frac{P-L}{P}\times 100$$ (11)

$$\text{RCR}=\frac{P}{L}$$ (12)

Permeabilized muscle fibers from the same gastrocnemius sample were run in duplicate across two O2k instruments (i.e., one duplicate chamber in each instrument); Data were excluded if either negative fiber bundle background respiration values following correction for instrumental background and/or low responsiveness to ADP titration (i.e., bundles did not respire in response to ADP (Bellissimo et al., 2023)) occurred. Only one of two duplicate values were included for 12 control samples and two *db/db* samples (i.e., at least one replicate is included from every biological sample/mouse).

Six control muscle samples had LEAK, ATP-linked, and CE (%) excluded from analysis due to erroneous addition of FCCP before oligomycin prior to a protocol change. Three control samples were excluded from analysis due to >20% increase in respiration after cytochrome c (CytC) addition; this cutoff is only slightly above general consensus values for exclusion and may indicate compromised mitochondrial membrane integrity (Kuznetsov et al., 2008; Kuang et al., 2022). *db/db* CytC response is discussed below; we did not employ a CytC cutoff (Kuznetsov et al., 2004; Laner et al., 2014; Gnaiger, 2020a). Electron transport capacity (E) and ResCap measurements (data not shown) were excluded from correlation analysis due to the observation that FCCP did not induce maximal respiration rates, concordant with previous literature on murine skeletal muscle respirometry (E ≤ P in murine skeletal muscle) (Gnaiger, 2020b; Aragonés et al., 2008).

#### 2.4.7 Complex IV activity

Electron transport chain complex IV (CIV; cytochrome c oxidase) activity was assessed in the hearts of control and *db/db* mice via microplate enzyme activity assay, per the manufacturer’s instructions. Briefly, previously liquid nitrogen (LN_2_)-frozen hearts (n = 2-3 per group) were pulverized under LN_2_ and extracted using reagents provided by the manufacturer and subsequently applied to a 96-well plate containing pre-bound monoclonal antibody to CIV, concentration-matched at 0.25 mg/mL (n = 8 technical replicates per heart sample). Activity of CIV (the oxidation of reduced CytC) in the presence of saturating CytC was measured as an absorbance change at 550 nm (expressed as mOD/min) in a Spectramax plate reader. Rate data were transformed and expressed as activity relative to control.

### 2.5 Quantification and statistical analysis

#### 2.5.1 Statistics

Statistical analyses were conducted in GraphPad Prism (La Jolla, CA, United States). We performed an *a priori* power analysis (G*Power, test family *Exact*, ‘Correlation: Bivariate normal model’) assuming a medium anticipated effect size (r = 0.50), two-tailed α = 0.05, and 1–β (power) = 0.80, which indicated a total sample size of 28. We targeted n = 30 to accommodate potential data loss. Applying the same parameters listed, but adjusting sample size to reflect the addition of n = 12 *db/db* mice, a post-hoc calculation indicated 1–β (power) = 0.93 for correlation analyses. Each respiration parameter was assessed for normality by Shapiro-Wilk test; only XF maximal respiration deviated from normality. Outliers were determined via ROUT outlier analysis (Q = 1%) and excluded. Group differences were assessed via two-tailed students t-tests with Welch’s correction for normal data and via Mann-Whitney test for nonparametric data. Correlations were computed using Spearman’s ρ for non-normal data and Pearson’s r for normally distributed data, with two-tailed p-values and 95% confidence intervals. Results were considered significant at p < 0.05.

## 3 Results

### 3.1 Murine platelet HRR

**FIGURE 1:**
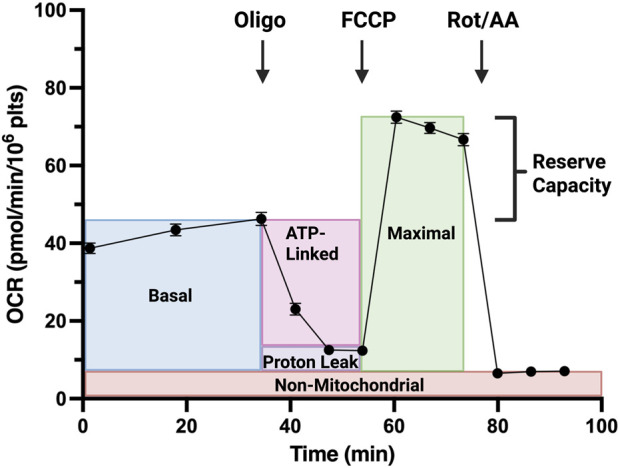
Mitochondrial Stress Test performed on platelets of control mice (n = 23 ± SEM). Effector injections are indicated by black arrows. Respiration parameters are indicated on the trace. *Oligo–Oligomycin; FCCP - Carbonyl cyanide-p-trifluoromethoxyphenylhydrazone; Rot/AA–Rotenone/Antimycin A*.

We have optimized a protocol for executing the Agilent Mitochondrial Stress Test in murine platelets using the Seahorse Extracellular Flux Analyzer (XFe24). Grouped platelet kinetic data from the XFe24 are displayed in Figure 1. Human platelet respirometry has been performed by numerous groups (Wilkinson and Dunham-Snary, 2023; Braganza et al., 2020; Braganza et al., 2019; Rose et al., 2019), with methodological guidelines published (Braganza et al., 2020; Chacko et al., 2013). However, special considerations must be taken when handling murine samples to account for differences between human and murine platelets (Jirouskova et al., 2007) that may impact bioenergetic assay execution.

### 3.2 Handling murine blood and platelet samples

The average circulating blood volume of an adult mouse is 72 mL/kg (Diehl et al., 2001) and the average 6-week-old C57BL/6J male mouse weighs 21.7 g (JAX, 2025); therefore, the total circulating blood volume of a 6-week-old male C57BL/6J mouse is ∼1.6 mL. Due to this small circulating blood volume, on average, approximately 1 mL of whole blood can be obtained per mouse. Collection of blood into a pre-loaded syringe containing sodium citrate prevents coagulation and minimizes platelet activation (Hechler et al., 2019). When isolating platelets from whole blood samples via centrifugation, care while pipetting is essential to avoid contamination of leukocytes from the buffy coat layer that resides under the PRP. After maximal collection of PRP, addition of platelet buffer and re-centrifugation of whole blood allows for a second collection of the uppermost layer, containing any leftover PRP while avoiding leukocyte contamination. By performing two centrifugation steps, volume of PRP obtained is maximized before pelleting and washing platelets with erythrocyte lysis buffer, which removes contaminating red blood cells. During these steps, presence of PGI_2_ in the platelet buffer prevents platelet activation and aggregation (Weiss and Turitto, 1979).

### 3.3 Cell seeding density

**FIGURE 2:**
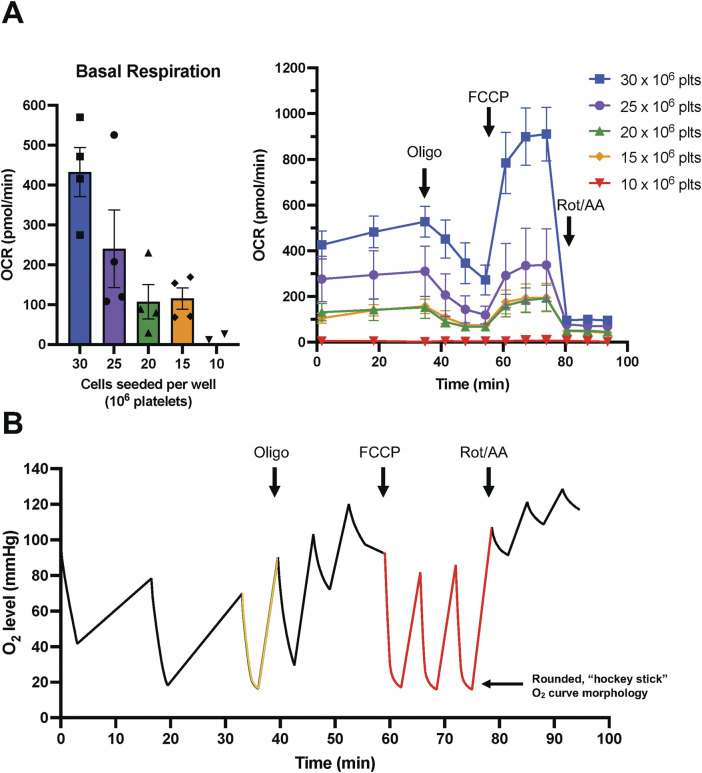
Murine platelet XFe24 assay optimization. **(A)** Cell seeding density assay. Each cell seeding density was tested with n = 4 replicate wells, ±SEM. Bar graph (left) depicts mean OCR_basal_ for increasing cell seeding densities. Kinetic trace (right) depicts mitochondrial stress test performed at various cell densities; effector addition is indicated by black arrows. **(B)** Oxygen (O_2_) level during platelet mitochondrial stress test indicating O_2_ and/or cellular exhaustion. n = 1 representative trace for 25 × 10^6^ platelets. The black line indicates O_2_ levels over time, with the yellow and red sections indicating areas of modest and overt O_2_ exhaustion, respectively. Effector addition is indicated by black arrows. *Oligo–Oligomycin; FCCP - Carbonyl cyanide-p-trifluoromethoxyphenylhydrazone; Rot/AA–Rotenone/Antimycin A*.

As with any XFe24 assay, cell seeding density must be taken into consideration. While seeding density guidelines have been validated for human platelets (Cardenes et al., 2014), we considered that differences in platelet size (Corash, 1989) and counting method (Walkowiak et al., 1997) applied to human versus mouse platelets may influence the optimal cell seeding density. After performing a cell seeding density assay (Figure 2A), platelets were initially seeded at a density of 25 × 10^6^ per well based on optimal OCR_basal_ values (Agilent recommends OCR_basal_ of 50–400 pmol/min for the XFe24 (Agilent Technologies, 2017)). While we achieved stable respiration rates within the recommended range with different cell densities, we documented a change in oxygen level morphology in response to FCCP at higher cell densities (Figure 2B). This J-shape morphology in the oxygen level trace, coupled with oxygen levels approaching 0 mmHg, indicate that the cells have exhausted the oxygen in the measurement chamber, and are unintentionally becoming hypoxic (Osto et al., 2020). Seeding density was reduced to 20 × 10^6^ platelets per well to avoid intermittent hypoxia/anoxia. ‘In-house’ titration of seeding density is recommended prior to commencing large-scale studies, as lower densities (e.g., 15 × 10^6^ platelets) will increase the likelihood that additional sample will be available for duplicate well analysis and allows for large increases in OCR in response to FCCP without risk of exhausting oxygen levels.

### 3.4 XFe24 plating technique and other assay considerations

Because platelets are plated in suspension, they are handled differently than adherent cells; centrifugation of the plate evenly distributes platelets across the bottom of each well, without the need to coat the plate with agents that improve cell adhesion like Cell-Tak or Matrigel. Care must be taken when adding media to avoid disturbing the platelet layer at the bottom of each well. Volumes smaller than a typical assay in a XFe24 are employed: a total well volume of 225 µL and injection volumes in port A, B, and C at 25 μL, 27.5 µL, and 31, µL, respectively, minimize ‘flushing’ the wells and lifting the cells. Once media is added to the cell plate, only 30 min of degassing is required before assay commencement. Leaving the cells for longer than 30 min may result in unstable respiration data; if unavoidable, data should be assessed for inclusion based on stability of OCR_basal_ across measurement cycles, expected Mitochondrial Stress Test kinetic trace morphology, and appropriate oxygen level morphology (Osto et al., 2020). Finally, as seen in the extended basal measurement section of the bioenergetic trace (Figure 1), we employ a 10-min wait period between the 3-min mix and 3-min measure periods during baseline measurements, whereas post-injection measurements have a wait time of 0 min. This is to ensure a stable baseline is achieved before assessing platelet response to effector addition. If any slight mechanical activation of the platelets is occurring during those initial basal measurements, this may be reversible (De Simone et al., 2023) during the extended wait period allowing for an accurate representation of the basal metabolism of resting platelets.

### 3.5 *db/db* mice exhibit increased mitochondrial respiration rates in platelets

**FIGURE 3:**
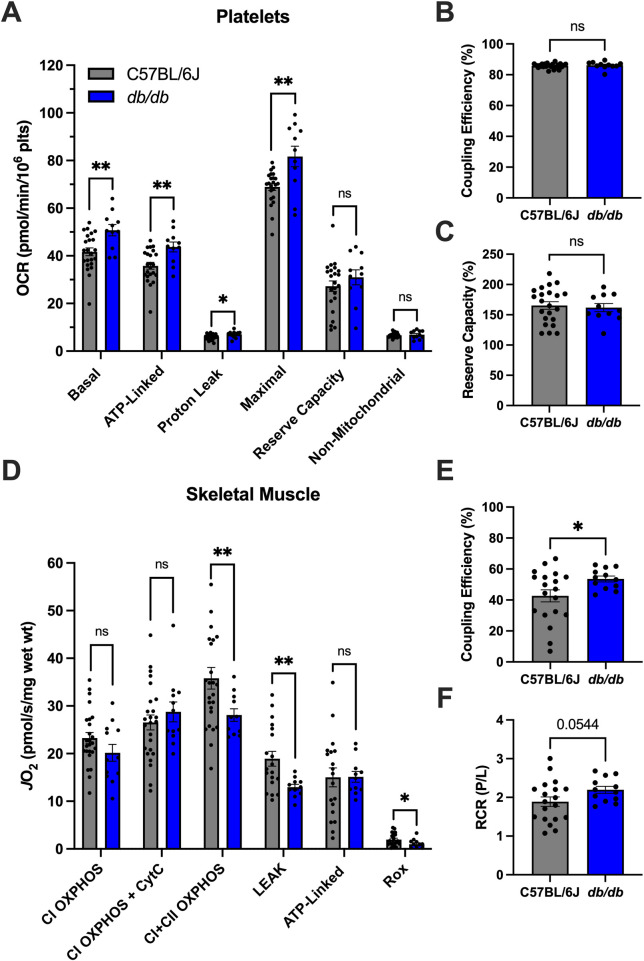
Platelet and gastrocnemius mitochondrial respiration (mean ± SEM). **(A)** Platelet oxygen consumption rate (OCR) of C57BL/6J (grey) and *db/db* (blue) mice. Values were normalized to platelet seeding density. **(B)** Platelet coupling efficiency (CE (%)). **(C)** Platelet reserve capacity (ResCap) (%). **(D)** Gastrocnemius muscle fiber oxygen flux (*J*O_2_) of C57BL/6J (grey) and *db/db* (blue) mice. Values were normalized to fiber bundle wet weight (wt). **(E)** Muscle coupling efficiency (CE (%)). **(F)** Muscle respiratory control ratio (RCR). * – p < 0.05; ** – p < 0.01; ns–not significant.

Platelet mitochondrial bioenergetics reveal differences in metabolism between C57BL/6J control and *db/db* mice (Figure 3). OCR_basal_ was significantly higher in *db/db* platelets relative to controls (p = 0.005; mean difference 9.066 ± 2.878 pmol/min/10^6^ platelets, Figure 3A), due to an increase in OCR_ATP_ (p = 0.005; mean difference 7.923 ± 2.524 pmol/min/10^6^ platelets, Figure 3A). Despite *db/db* platelets exhibiting a significant gain in OCR_leak_ (p = 0.035; mean difference 1.136 ± 0.4938 pmol/min/10^6^ platelets, Figure 3A), which relates to mitochondrial coupling state, CE (%) did not differ between *db/db* and control mice (p = 0.63; mean difference 0.3777% ± 0.7784%, Figure 3B) due to concomitant increases in both OCR_basal_ and OCR_ATP_ (the parameters from which CE (%) is derived).

OCR_max_ was also increased in *db/db* platelets relative to controls (p = 0.006; mean difference 14.12 pmol/min/10^6^ platelets, Figure 3A), but there was no significant difference found in ResCap expressed as the difference between OCR_max_ and OCR_basal_ (Figure 3A) or as a percent of OCR_basal_ (Figure 3C). By accounting for OCR_basal_, ResCap is a measure of the cells’ ability to respond to increased energy demand or under stress (Divakaruni et al., 2014).

### 3.6 *db/db* mice exhibit impaired skeletal muscle mitochondrial metabolism

Murine skeletal muscle respirometry in white gastrocnemius muscle was performed using the Oxygraph-2 k (O2k; Oroboros), performing a substrate-uncoupler-inhibitor-titration (SUIT) protocol that mimics the Agilent Mitochondrial Stress Test assay, but FCCP measurements were excluded (as previously discussed in Methods) in accordance with previous literature on murine skeletal muscle respirometry (Gnaiger, 2020b; Aragonés et al., 2008). Mitochondrial respiration parameters obtained from skeletal muscle via the O2k include CI OXPHOS, CI + CII OXPHOS (P), LEAK (L), ATP-linked, CE (%), respiratory control ratio (RCR), and residual oxygen consumption (Rox). We have also included CI OXPHOS + CytC as this step occurred before addition of succinate, to better reflect true CI + CII OXPHOS without bias from response to CytC addition. This is most relevant for *db/db* samples which had a larger response to CytC and minimal respiration gain in response to succinate addition. In addition to the traditional respiratory parameters discussed, we also explored flux control efficiency (FCE) ratios (Gnaiger, 2020a) for both CytC and succinate, as well as absolute gain in oxygen flux, to assess the impact of each substrate on respiration in isolation (Supplementary Figure S4). Briefly, *db/db* muscle had a significantly larger absolute increase in oxygen flux when exogenous CytC was added (p = 0.003, mean difference 5.337 ± 1.517 pmol/s/mg wet wt, Supplementary Figure S4A) and a blunted absolute response to succinate addition (p < 0.0001, median difference −5.558 pmol/s/mg wet wt, Supplementary Figure S4B) compared to control skeletal muscle. The same pattern was observed in FCE ratios, whereby *db/db* muscle had increased CytC FCE (p = 0.0009, mean difference 0.1932 ± 0.04762, Supplementary Figure S4C) and decreased succinate FCE (p < 0.0001, median difference −0.1756, Supplementary Figure S4D) relative to control muscle. Lack of significant difference between control and *db/db* CI OXPHOS and CI OXPHOS + CytC, paired with significant differences in CI + CII OXPHOS (Figure 3D), indicates that CytC release in *db/db* muscle does not impact interpretation of results, and was not the reason for later deficits in *db/db* muscle respiration. Further, supplementary analysis of the absolute succinate response and S-pathway FCE reveal a CII-linked deficit in *db/db* tissue independent of CytC response. To visualize potential deficits in CIV in our study animals, we conducted a CIV activity assay using control and *db/db* hearts (Supplementary Figure S5) and found a ∼20% reduction in CIV activity in *db/db* hearts (p < 0.0001), supporting the conclusion that the larger response to exogenous CytC in *db/db* muscle reflects the underlying disease, and not a technical artefact of the tissue preparation.

Skeletal muscle respirometry revealed metabolic patterns in *db/db* mice that followed opposing directionality to our findings in platelets. While *db/db* muscle fibers did not differ from C57BL/6J mice when measuring OXPHOS driven by complex I substrates, there was a deficit in OXPHOS supported by both complex I and II substrates (p = 0.006; mean difference −7.722 ± 2.634 pmol/s/mg wet wt, Figure 3D). Additionally, L was reduced in *db/db* skeletal muscle relative to control mice (p = 0.002; mean difference −5.955 ± 1.662 pmol/s/mg wet wt, Figure 3D). Due to this reduction in L and lack of change in ATP-linked respiration (Figure 3D), *db/db* muscle had significantly increased CE (%) (p = 0.019; mean difference 10.90% ± 4.339%, Figure 3E) compared to C57BL/6J and a trending increase in RCR (p = 0.054; mean difference 0.3061 ± 0.1527, Figure 3F). As well, Rox was significantly lower in *db/db* muscle (p = 0.027; mean difference 0.9542 ± 0.4084 pmol/s/mg wet wt, Figure 3D).

### 3.7 Correlation of platelet and muscle respirometry

We have discovered multiple physiologically relevant correlations between platelet and skeletal muscle bioenergetics in both healthy male C57BL/6J and diabetic *db/db* mice demonstrating the opposing pattern of bioenergetic adaptations to murine T2DM observed in platelets and skeletal muscle.

**FIGURE 4:**
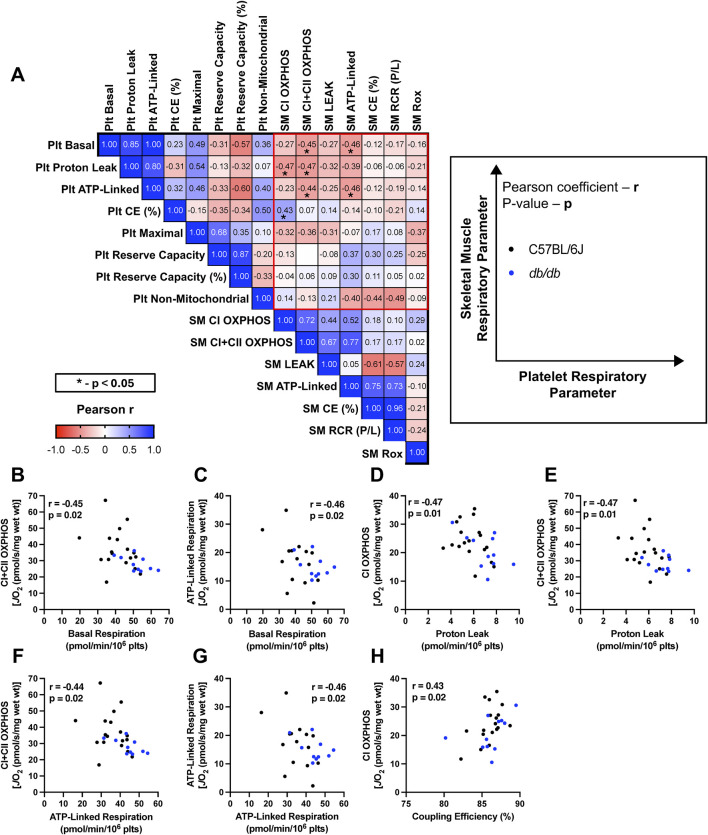
Correlations between platelet (Plt) and skeletal muscle (SM) mitochondrial respiratory states from control and db/db mice. **(A)** Correlation matrix of respiratory states (left). Blue and red represent positive and negative Pearson correlation coefficient (r) values, respectively. Color saturation indicates the strength of correlation. Example correlation graph for ease of interpretation (right). **(B−H)** Individual Pearson correlation graphs of Plt and SM respiratory parameters. SM (y-axes) and Plt (x-axes) respiration data were normalized to tissue wet weight (wt) and cell seeding density, respectively. Pearson r and p values are reported on each graph. **(B)** Plt basal respiration and SM CI + CII OXPHOS. **(C)** Plt basal respiration and SM ATP-linked. **(D)** Plt proton leak and SM CI OXPHOS. **(E)** Plt proton leak and SM CI + CII OXPHOS. **(F)** Plt ATP-linked respiration and SM CI + CII OXPHOS. **(G)** Plt ATP-linked respiration and SM ATP-linked respiration. **(H)** Plt CE (%) and SM CI OXPHOS. CE (%) – % Coupling Efficiency; RCR–Respiratory Control Ratio; Rox–Residual Oxygen Consumption.

When assessing the entire cohort of C57BL/6J and *db/db* mice, numerous correlations were found between platelet and skeletal muscle mitochondrial bioenergetic parameters (Figure 4). Platelet OCR_basal_ negatively correlates with skeletal muscle CI + CII OXPHOS (Figure 4B), demonstrating concomitant, opposing changes in platelet and skeletal muscle baseline respiration. Basal respiration was increased in *db/db* mice relative to control; CI + CII OXPHOS was reduced in *db/db* relative to control. Platelet OCR_basal_ also correlated with muscle ATP-linked respiration (Figure 4C), which was unchanged in *db/db* relative to control; while basal respiration provides an informative starting point for linking platelet and muscle bioenergetics, it represents a composite of several mitochondrial and non-mitochondrial processes and can vary with intrinsic substrate availability. Future investigations focusing on coupling efficiency (which offers a more stable and physiologically relevant metric of mitochondrial control in intact cells) would further strengthen comparisons between assays and platforms, and could better standardize interpretation of findings.

OCR_basal_ can be segregated into OCR_ATP_ and OCR_leak_ through inhibition of ATP synthase; platelet OCR_leak_ correlated with CI OXPHOS (Figure 4D) and CI + CII OXPHOS (Figure 4E). Platelet OCR_ATP_, which was inflated in *db/db* mice, correlated with both CI + CII OXPHOS (Figure 4F) and muscle ATP-linked respiration (Figure 4G). Although CE (%) did not differ between control and *db/db* platelets, platelet CE (%) correlated positively with skeletal muscle CI OXPHOS (Figure 4H). Interestingly, these parameters correlate both when considering the controls alone, as well as when analyzing control and *db/db* samples together in a single cohort (as shown in Figure 4H). This correlation links platelet coupling state and muscle complex I function in health and disease. In a context-dependent manner, mitochondrial (un)coupling can support biologically protective processes including buffering during ROS-favouring conditions or can also indicate global mitochondrial dysfunction (Demine et al., 2019).

## 4 Discussion

We have described comprehensive methodological and optimization guidelines for assessing platelet bioenergetics in mice, as well as reported novel observations in a murine model of T2DM. While some groups report platelets from human T2DM patients exhibit decreased respiration values compared to controls (Avila et al., 2012; Gao et al., 2022), and this decrease can be related to markers of oxidative stress (Avila et al., 2012), we have demonstrated the opposite in murine T2DM. Our findings demonstrate increased respiration in *db/db* platelets relative to healthy controls (Figure 3), with conserved CE (%) and ResCap. Siewiera et al. (2022) have also reported that diabetic rats have *increased* ROUTINE respiration in intact platelets compared to non-diabetic controls and a trending increase in LEAK respiration. They further showed that metformin treatment attenuates these elevations in diabetic rat platelet respiration, which may help explain why human studies often report reduced respiration values in diabetic patients versus controls. Since it would be neither feasible nor ethically acceptable to withdraw standard treatments from T2DM patients to interrogate platelet metabolism, animal models may capture changes that are masked in human cohorts receiving drug therapy. Avram et al. (2021) report no difference in platelet respiration in T2DM patients treated with or without statin compared to healthy controls. However, untreated T2DM patients exhibited non-significant increases in platelet respiration in this study, consistent with findings from animal models of diabetes.

While numerous analyses of muscle bioenergetics in animal models of obesity and diabetes are documented, results are conflicting. Adult Zucker rats exhibit impaired respiration in mitochondria isolated from soleus muscle that is not present in young rats (Hey-Mogensen et al., 2012). Conversely, obese Wistar rats exhibit increased OXPHOS and ET capacities in diaphragm muscle compared to lean controls (Rodrigues et al., 2020). In mitochondria isolated from cardiac muscle of obese C57BL/6J mice, Boardman et al. (2020) demonstrated decreased pyruvate- and palmitate-stimulated state 3 and state 4 respiration compared to age-matched controls, with no change in RCR. As RCR is calculated by state 3/state 4 (OXPHOS/LEAK), the decrease in OXPHOS without a change in RCR is due to the concomitant decrease in state 4 respiration. Similar results were demonstrated in isolated cardiac mitochondria of obese Zucker rats, exhibiting decreased state 3 and state 3u respiration compared to lean controls (Guarini et al., 2016). When assessing skeletal muscle in animal models of diabetes, respiration trends from extensor digitorum versus soleus muscle show conflicting patterns; LEAK, CI-linked OXPHOS, CI + CII-linked OXPHOS, and ET capacity are increased in extensor digitorum muscle of *db/db* mice, but CI-linked OXPHOS is decreased in soleus muscle (Holmström et al., 2012), supporting the finding that diabetes differentially impacts mitochondrial metabolism in skeletal muscle subtypes (Rabøl et al., 2010). We demonstrate deficits in CI + CII-linked OXPHOS, LEAK, and Rox in white gastrocnemius of *db/db* mice. When comparing tibialis anterior muscle of lean non-diabetic *fa*/+ and obese diabetic *fa*/*fa* adult male Zucker diabetic fatty rats, Wessels et al. (2014) found no differences in CI- or CII-linked OXHPOS, but increased CI-linked RCR in diabetic animals suggesting uncoupling. An interesting avenue of further investigation is assessing the applicability of platelet bioenergetics predictive ability to a mixed fiber type (e.g., vastus lateralis) or predominantly type I, “slow twitch” fiber muscle (e.g., soleus) metabolism, as the work herein was performed in a predominantly type II, “fast twitch” fiber.

The *db/db* skeletal muscle respiration data presented in Figure 3 was not subject to a CytC response cutoff, and all muscle fiber bundles harvested from *db/db* mice exhibited increased respiration in response to exogenous CytC (added after assessing CI-linked OXPHOS, but before capturing response to succinate). While the literature lacks formal consensus for the inclusion/exclusion of respiration data with a “larger” CytC response, an elevated response to exogenous CytC is not unexpected in a severe disease state such as that present in the *db/db* mouse: Pinti et al. (2019) have reviewed the global effects of altered lipid metabolism and overproduction of mitochondrial ROS in both *db/db* mice and Zucker diabetic fatty rats, linking mitochondrial ROS and organelle integrity to continuous mitochondrial maladaptation throughout T2DM progression; both Sivitz and Yorek (2010) and Belosludtsev et al. (2020) extensively reviewed mitochondrial dysfunction in diabetic muscle, noting increased membrane damage and altered permeability as hallmarks of the disease, which would amplify the effect of exogenous CytC; Picard et al. (2011) acknowledge that interpreting CytC response in pathologic muscle (aging, chronic disease, etc.) can be confounded by pre-existing membrane instability; and Kuznetsov et al. (2004) note that heterogeneous mitochondrial damage is characteristic for various pathologies including CytC release, indicating inherent mitochondrial fragility. We acknowledge that the permeabilization protocol *may* have induced additional damage to an already fragile mitochondrial membrane, but the more likely explanation is that we captured another aspect of the murine T2DM phenotype. While Mootha et al. (2001) report that the addition of exogenous CytC to Fas-activated hepatocytes helped restore respiratory function, we did not observe significant differences in complex I-linked respiration between control and *db/db* mice when exogenous CytC was added (CI OXPHOS and CI OXPHOS + CytC, Figure 3D). As downstream measurements in the presence of saturating CytC are conducted after reconstitution of the N pathway, we subsequently observed both a CytC-related injury inherent to *db/db* muscle as well as a CII-linked deficit in *db/db* muscle. Further, this CytC-related injury was recapitulated in the heart muscle of these animals; the activity of ETC CIV was dramatically reduced in the hearts of *db/db* mice compared to controls (Supplementary Figure S5), indicating that mitochondrial and ETC fragility is present across multiple tissues in this mouse model. Lastly, we also note that inclusion of this disease model increased the strength of correlations observed (i.e., the correlations are more robust when considering control + *db/db* as a single cohort than in control alone).

This report is the first to assess the correlation between platelet and skeletal muscle mitochondrial bioenergetics in mice, and to demonstrate the utility of platelet bioenergetics as a surrogate for skeletal muscle bioenergetics in healthy mice and a murine model of T2DM. Correlations with skeletal muscle CI + CII OXPHOS and ATP-linked respiration (Figure 4) indicate that platelet bioenergetics are a reliable surrogate for skeletal muscle respiration in preclinical T2DM. As platelet OCR_basal_ and OCR_ATP_ increase, we have demonstrated that CI + CII OXPHOS and ATP-linked respiration in skeletal muscle decrease (Figure 4). Therefore, when platelets exhibit a pathogenic augmentation of basal respiration linked to ADP phosphorylation, the opposite is occurring in skeletal muscle. As well, our finding that platelet CE (%) positively correlates with muscle CI OXPHOS (Figure 4H) guides a subsequent hypothesis that the coupling state of platelets may relate to skeletal muscle complex I function; when platelets are less coupled, the skeletal muscle has a lower respiration driven by complex I substrates. Therefore, we propose that minor perturbances in the highly conserved coupling state observed in platelet mitochondria may reflect variability in complex I-driven respiration in muscle of the same animals. The mechanism of this platelet-muscle connection is of tremendous value, but beyond the scope of this brief research report, and further investigation will be required.

While preclinical models of obesity and T2DM have translational limitations, the skeletal muscle phenotype in humans is well-documented. In human obesity, Vijgen et al. (2013) have shown that skeletal muscle of morbidly obese patients has decreased CI + CII-linked OXPHOS capacity, with unchanged LEAK respiration compared to lean controls. Further, they found that weight loss induced 1 year after bariatric surgery diminished the contribution of LEAK to OXPHOS. As well, Phielix et al. (2014) demonstrated that obese individuals have decreases in CI- and CI + CII-linked OXPHOS, as well as ET capacity compared to lean controls. These findings contrast work from Ara et al. (2011) who reported that obesity did not induce changes in skeletal muscle mitochondrial respiration compared to lean or post-obese controls; they report a trending increase in CI-linked OXPHOS in muscle from obese participants after respiration data was normalized to citrate synthase activity. Similarly, when investigating the impact of “pre-diabetes” on skeletal muscle respiration, Szczerbinski et al. (2021) found no change in any mitochondrial respiration parameters before or after exercise intervention. The findings from Ara et al. (2011) are somewhat incongruent with the current understanding of skeletal muscle metabolic adaptations to exercise (Lundby and Jacobs, 2016), therefore, further investigation into how the “pre-diabetes state” influences skeletal muscle mitochondrial respiration is warranted. It is well documented that diabetes confers a deficit in skeletal muscle respiration (Wessels et al., 2014; Phielix et al., 2014; Antoun et al., 2015; Avram et al., 2022; Larsen et al., 2009; Mogensen et al., 2007; Rabøl et al., 2009); an inverse relationship between HbA1c and mitochondrial respiration parameters in both T2DM patients and non-diabetics has been demonstrated (Antoun et al., 2015; Mogensen et al., 2007). An additional consideration to future investigations is the inconsistency in respirometry from a conceptual perspective, highlighting the need for robust biomarkers of inaccessible cells and tissues.

Human studies investigating platelet bioenergetics have yielded promising early results, but correlation between skeletal muscle and platelet mitochondrial respiration has yet to be assessed in CMD patients. When comparing platelet and muscle bioenergetics of healthy women of various BMI status, Rose et al. (2019) report that CI-linked LEAK respiration and OXPHOS coupling efficiency both positively correlate between the tissues. As well, Braganza et al. (2019) showed correlation between muscle and platelet bioenergetics in young versus older adults. However, Westerlund et al. (2024) found few significant correlations between platelet and skeletal muscle metabolism in athlete and non-athlete controls; they revealed negative correlations in background respiration and NS-OXPHOS capacity. They also demonstrated that augmented mass-specific muscle respiration shown in athletes was not reflected in platelet metabolism but observed significantly altered platelet bioenergetics in a subset of patients with primary mitochondrial disease. Correlation between platelet and skeletal muscle bioenergetics has also been demonstrated in non-human primates, specifically, African green monkeys (Tyrrell et al., 2016). It is worth noting that sex-based differences in platelet bioenergetics may be present – Mahapatra et al. (2024) report higher respiration rates in female platelets as compared to males. Further research is necessary to explore the potential of platelet bioenergetics as a muscle-specific mitochondrial biomarker and should include both male and female participants.

The work described herein establishes novel correlations between platelet and skeletal muscle bioenergetics in a murine model of T2DM, demonstrating that platelet bioenergetics may serve as a surrogate for skeletal muscle mitochondrial health. These findings provide a foundation for future work investigating how platelet bioenergetics respond to metabolic challenges, such as nutrient overload.

## 5 Conclusion

Platelets have the potential to serve as a skeletal muscle-specific bioenergetic marker and this proof-of-concept study lays the groundwork for studying the impact of other genetic, epigenetic, and environmental factors on both platelet and muscle bioenergetics in animal models, as well as for future human studies in healthy individuals and metabolic disease patients. We demonstrated correlation between the measures of interest in healthy mice and a murine model of T2DM, warranting further investigation into this marker in the context of metabolic diseases. If platelet mitochondria can reflect early metabolic changes signaling the onset of complex metabolic diseases, especially *before* perturbations in traditional clinical markers, this method may, in the future, serve as a potent clinical tool for streamlined detection of pathology and implementation of prevention and intervention strategies before the onset of overt disease.

## Data Availability

The raw data supporting the conclusions of this article will be made available by the authors, without undue reservation.
