# Supplementary material for: Platelet bioenergetics correlate with skeletal muscle respiration in a murine model of type II diabetes
Source: Front Mol Biosci. 2025 Nov 14;12:1639882. doi: 10.3389/fmolb.2025.1639882 (PMC12660101; doi:10.3389/fmolb.2025.1639882)
Supplement: Supplementary file 1 [file DataSheet1.docx]

Supplementary Material

# Supplementary Figures and Tables

## Supplementary Figures


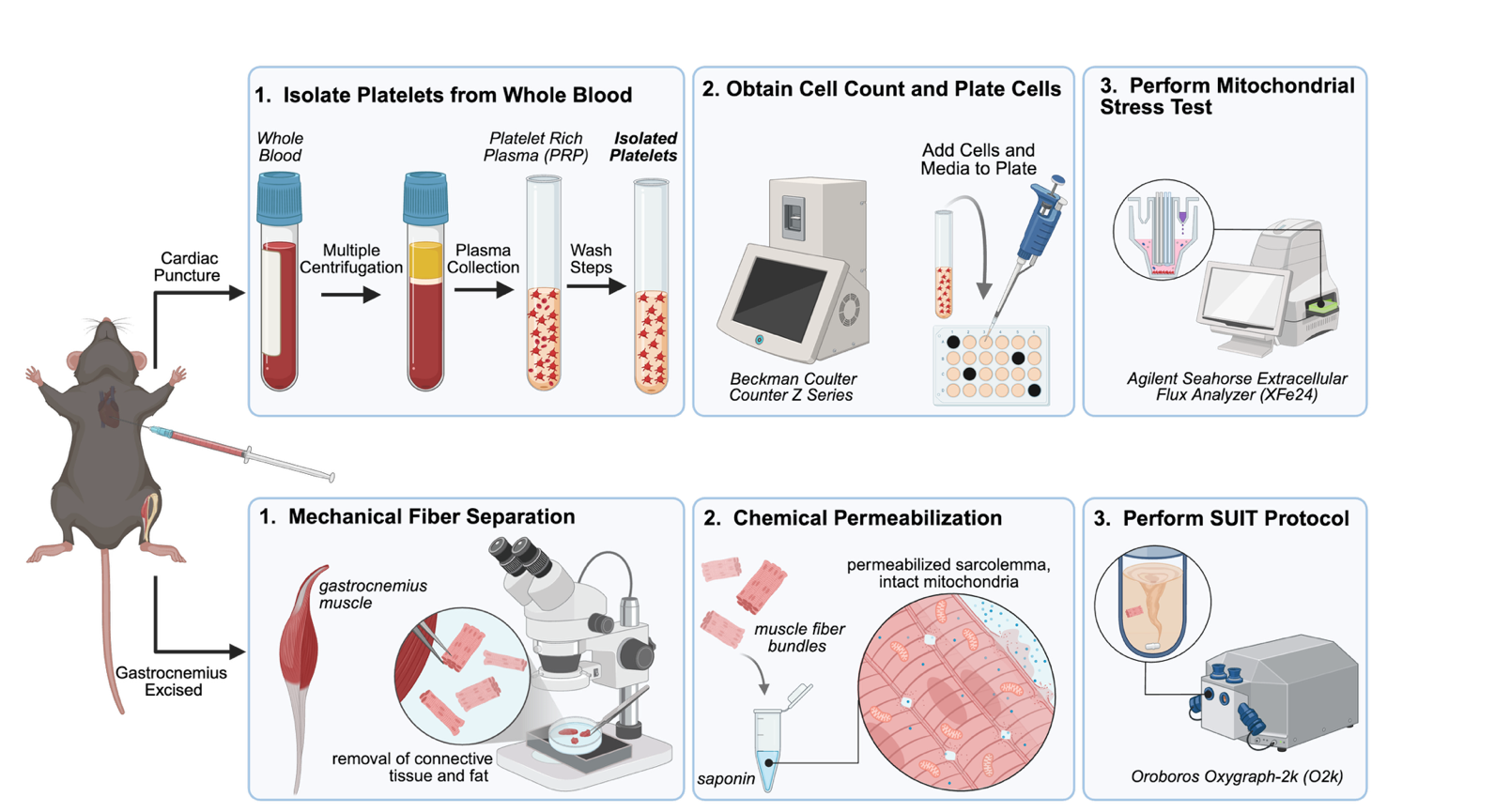


**Supplementary Figure 1. Experimental workflow.** After collection of whole blood and gastrocnemius muscle, tissue preparations must be performed before HRR assays commence to yield isolated platelets and permeabilized muscle fiber bundles, respectively. Platelet and muscle processing steps are shown in the top and bottom panels, respectively. *SUIT – Substrate-uncoupler-inhibitor titration.*

**Supplementary Figure 2.** **Assessment of platelet isolation purity.** **A.** CD41 positive cells (platelets) from three murine samples demonstrating the purity of the platelet isolates. Percentage of total events is indicated on each graph with decimals denoted with commas. **B-C.** CD45 (leukocyte marker) negative (left) and positive (right) events in quiescent (**B**) and thrombin-activated (**C**) platelet isolates demonstrating purity of the platelet samples. Percentage of total events is indicated on each graph with decimals denoted with commas.

**Supplementary Figure 3.** **Assessment of platelet isolation activation status.** P-selectin positive cells (activated platelets) in unstimulated platelet isolates and platelets incubated with thrombin (a platelet activator) demonstrating that the platelet isolation protocol itself does not activate platelets. Percentage of total events for the quiescent samples is indicated on each graph with decimals denoted with commas.


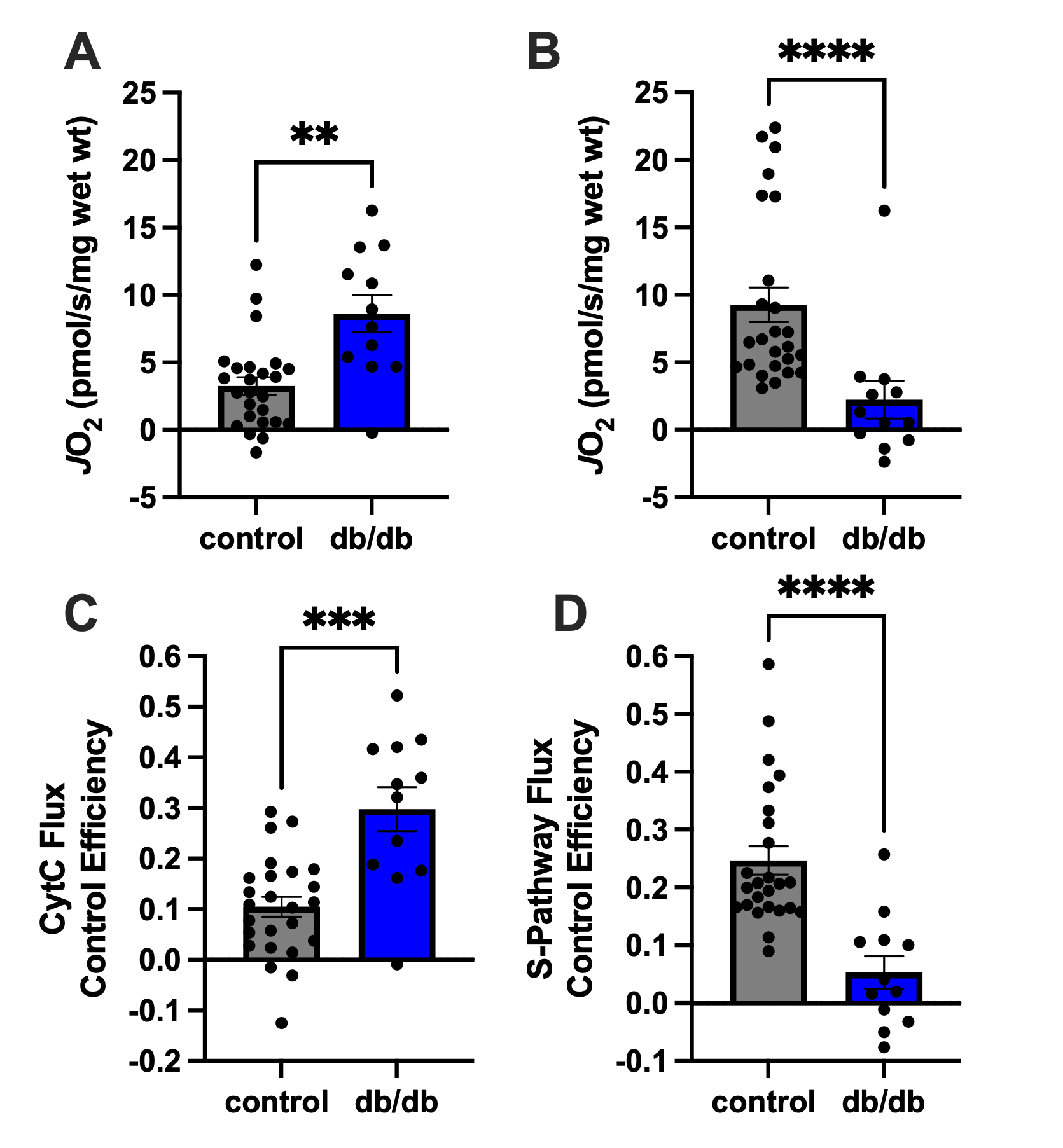


**Supplementary Figure 4.** **Cytochrome C (CytC) and S-pathway respiratory analysis in gastrocnemius muscle of control and db/db mice (mean ± SEM). A.** Absolute increase in oxygen flux upon exogenous CytC addition. **B.** Absolute increase in oxygen flux upon exogenous succinate addition. **C.** CytC flux control efficiency calculated as (CI-OXPHOS+CytC – CI-OXPHOS) / CI-OXPHOS+CytC. **D.** S-pathway flux control efficiency calculated as (CI+CII-OXPHOS – CI-OXPHOS+CytC) / CI+CII-OXPHOS. *** – p < 0.01, *** – p < 0.001, **** – p < 0.0001.*


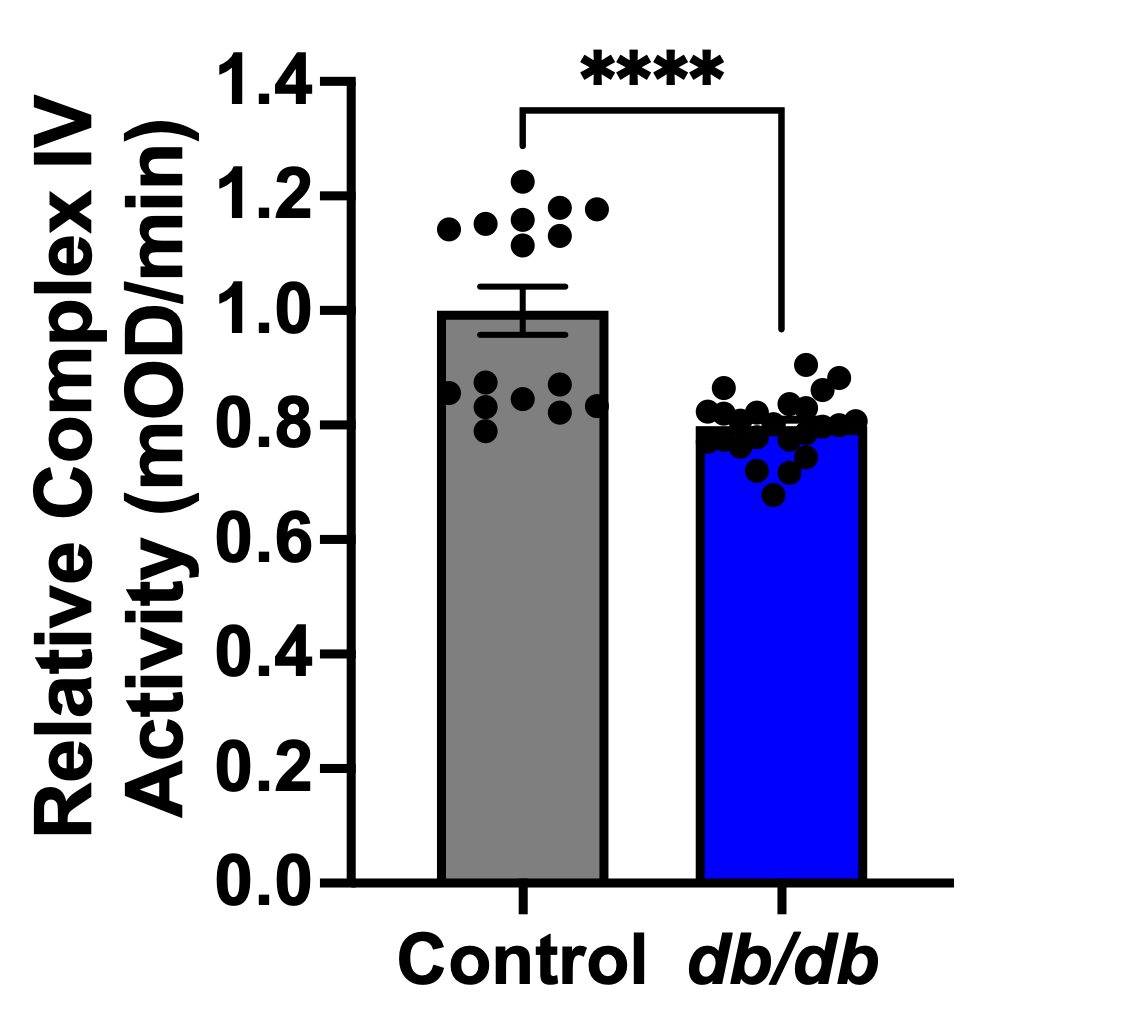


**Supplementary Figure 5.** **Relative Complex IV activity of control (grey bar) and *db/db* (blue bar) heart tissue (mean ± SEM).** Data points represent technical replicates from n = 2-3 mice per group. Relative complex IV activity (x-axis) is expressed as the average milli-optical density/minute determined by kinetic plate assay. ***** – p < 0.0001.*
